# The epidemiology of bloodstream infections and antimicrobial susceptibility patterns in Thuringia, Germany: a five-year prospective, state-wide surveillance study (AlertsNet)

**DOI:** 10.1186/s13756-021-00997-6

**Published:** 2021-09-08

**Authors:** Franziska Schöneweck, Roland P. H. Schmitz, Florian Rißner, André Scherag, Bettina Löffler, Mathias W. Pletz, Sebastian Weis, Frank M. Brunkhorst, Stefan Hagel

**Affiliations:** 1 Center for Sepsis Control and Care (CSCC), Jena University Hospital – Friedrich Schiller University Jena, Jena, Germany; 2 Research Group Clinical Epidemiology, CSCC, Jena University Hospital – Friedrich Schiller University Jena, Jena, Germany; 3 Center for Clinical Studies Jena (ZKS), Jena University Hospital – Friedrich Schiller University Jena, Jena, Germany; 4 Institute of Medical Statistics, Computer and Data Sciences, Jena University Hospital – Friedrich Schiller University Jena, Jena, Germany; 5 Institute of Medical Microbiology, Jena University Hospital – Friedrich Schiller University Jena, Jena, Germany; 6 Institute for Infectious Diseases and Infection Control, Jena University Hospital – Friedrich Schiller University Jena, Am Klinikum 1, 07747 Jena, Germany; 7 Department of Anesthesiology and Intensive Care Therapy, Jena University Hospital – Friedrich Schiller University Jena, Jena, Germany

**Keywords:** Bloodstream infections, Surveillance, Epidemiology, MRSA, ESBL

## Abstract

**Background:**

Monitoring pathogens of bloodstream infections (BSI) and their antibiotic susceptibility is important to guide empiric antibiotic treatment strategies and prevention programs.

This study assessed the epidemiology of BSI and antibiotic resistance patterns at the German Federal State of Thuringia longitudinally.

**Methods:**

A surveillance network consisting of 26 hospitals was established to monitor BSIs from 01/2015 to 12/2019. All blood culture results, without restriction of age of patients, of the participating hospitals were reported by the respective microbiological laboratory. A single detection of obligate pathogens and a repeated detection of coagulase-negative staphylococci, *Bacillus* spp., *Corynebacterium* spp., *Micrococcus* spp. and *Propionibacterium* spp., within 96 h were regarded as a relevant positive blood culture. If one of the aforementioned non-obligate pathogens has been detected only once within 96 h, contamination has been assumed. Logistic regression models were applied to analyse the relationship between resistance, year of BSI and hospital size. Generalized estimating equations were used to address potential clustering.

**Results:**

A total of 343,284 blood cultures (BC) of 82,527 patients were recorded. Overall, 2.8% (n = 9571) of all BCs were classified as contaminated. At least one relevant pathogen was identified in 13.2% (n = 45,346) of BCs. *Escherichia coli* (25.4%) was the most commonly detected pathogen, followed by *Staphylococcus aureus* (15.2%), *Staphylococcus epidermidis* (8.1%) and *Klebsiella pneumoniae* (4.6%). In *S. aureus,* we observed a decline of methicillin resistance (MRSA) from 10.4% in 2015 to 2.5% in 2019 (*p* < 0.001). The rate of vancomycin resistance in *Enterococcus faecium* (VRE) has increased from 16.7% in 2015 to 26.9% in 2019 (*p* < 0.001), with a peak in 2018 (42.5%). In addition, we observed an increase of Cefotaxime (3GC) resistance in *E. coli* from 10.7% in 2015 to 14.5% in 2019 (*p* = 0.007) whereas 3GC resistance in *K. pneumoniae* was stable (2015: 9.9%; 2019: 7.4%, *p* = 0.35). Carbapenem resistance was less than 1% for both pathogens. These patterns were robustly observed across sensitivity analyses.

**Conclusions:**

We observed evidence for a decline in MRSA, an increase in VRE and a very low rate of carbapenem resistance in gram-negative bacteria. 3GC resistance in *E. coli* increased constantly over time.

**Supplementary Information:**

The online version contains supplementary material available at 10.1186/s13756-021-00997-6.

## Background

The term bloodstream infection (BSI) generally refers to the growth of a microorganism from a blood culture obtained from a patient with clinical signs of infection and where contamination has been ruled out [1]. BSIs are associated with a significant morbidity and mortality. Their incidence rate in population-based studies in North America and Europe ranges between 113 and 204 per 100,000 person-years [2]. BSIs comprise a wide variety of pathogens and clinical syndromes and may be either secondary to a focal infection including abscesses, pneumonia or urinary tract infections, or primary without another defined focus of infection [1]. The epidemiology of BSIs is influenced by several factors, e.g. demographic changes, the emergence of multi-drug resistant pathogens and advances in medicine with increasing numbers of immunocompromised patients and utilization of invasive devices [3, 4]. Continuously monitoring trends in the microbiology of BSI pathogens and their antibiotic susceptibility patterns is therefore important to guide empiric antibiotic treatment strategies and prevention programs, such as infection control measures or vaccination programs (e.g.; for pneumococci and *Haemophilus influenzae* B). This study aimed at describing the epidemiology of BSIs and antimicrobial resistance patterns for the clinically most relevant pathogens over a five-year period at the German Federal State of Thuringia.

## Methods

Surveillance for BSI was prospectively performed using the AlertsNet electronic blood culture registry (EBCR), whose protocol was previously published [5]. AlertsNet is a prospective surveillance study conducted in the German Federal State of Thuringia and a member of the international bacteremia surveillance collaborative (IBSC) since 2012 [6]. Thuringia is located in east-central Germany and has approximately 2.2 million inhabitants, 42 hospitals and approximately 740 beds per 100,000 population. Participation in the AlertsNet EBCR is voluntary. Overall, 26 hospitals have participated in the EBCR during the reported surveillance period from 01/2015 to 12/2019. Start date and duration of participation differed between hospitals. The number of hospital beds of participating hospitals varied between 59 and 1400 beds per hospital. Additional file 1: Table S1 (Supplement) provides an overview over hospital size and reporting period of each hospital.

All blood culture results, without restriction of age of patients, of the participating hospitals were reported by the respective microbiological laboratory to the EBCR. Information on pathogens, resistance patterns, age and sex of the patient were reported. Due to the fact, that commonly international standards of nomenclature for species names and anti-infectives are often not applied by the microbiological labs, individual parsing of the transmitted microbiological results had to be performed. One blood culture was definded as a set of one aerobic and one anaerobic blood culture bottle. A single detection of obligate pathogens and a repeated detection of coagulase-negative staphylococci (CoNS), *Bacillus* spp., *Corynebacterium* spp., *Micrococcus* spp. and *Propionibacterium* spp., within 96 h were regarded as a relevant positive blood culture, in the further analyses referred to as a “positive blood culture” [7]. If one of the aforementioned non-obligate pathogens has been detected only once within 96 h, contamination has been assumed.

An “episode” was defined as a positive blood culture with one pathogen. Multiple positive blood cultures with the same pathogen within 96 h were merged into one “episode” in the analyses. If the pathogen was detected again after 96 hs, a new “episode” was counted. An “event” was defined as a 96 h episode in which one *or* multiple positive blood cultures with the same or different pathogens were detected. For example, if one blood culture with *Candida albicans* and one blood culture with *Escherichia coli* were detected within 96 h, one episode of *C. albicans* BSI*,* one episode of *E. coli* BSI and one BSI event was recorded. Susceptibility results for *Pseudomonas aeruginosa* are presented only until 2018 because participating microbiological laboratories have implemented gradually the new European Committee on Antimicrobial Susceptibility Testing (EUCAST) breakpoints and methodology at different timepoints in 2019 [8]. Thus, an evaluation of antibiotic susceptibility test results between laboratories and different years was therefore no longer feasible for this pathogen. AlertsNet was approved by the Ethics Committee of the State Chamber of Physicians of Thuringia (Jena; 3 February 2014). The project protocol and technical IT concept were approved by the Thuringian State Commissioner for Data Protection and Freedom of Information (Erfurt, 10 April 2015, AZ: 278-7/2014.42).

### Statistical analyses

Standard descriptive statistics (absolute and relative frequencies; means/median and standard deviations and quartiles) were used to summarize categorical and continuous variables, respectively. We used logistic regression models to analyse the relationship between the (binary) medical resistance and the year of the BSI recording adjusting for hospital size (number of beds in categories). To address the potential clustering within the same hospitals, we applied generalized estimating equations (GEE). From these analyses, we report the unadjusted and hospital size-adjusted odds ratios (ORs) together with the 95% confidence intervals (CI) and corresponding two-sided *p* values (not adjusted for multiple testing). As additional sensitivity analyses to check the robustness of the regression results with regard to different analyses sets, we also performed the same analyses restricted to the 10 hospitals that contributed data for at least 36 months. Moreover, we also ran the corresponding mixed models on these data sets (with hospital as random intercept). As all these sensitivity analyses demonstrated consistent findings, we decided to omit them from this report. All analyses were conducted via R version 3.6.1 (R Development Core Team (2008). R: A language and environment for statistical computing. R Foundation for Statistical Computing, Vienna, Austria). For both, the GEE models and and for the mixed model we applied the gee R-package.

## Results

During the 5-year surveillance period, data from 82,527 patients (39.5% female, 4.2% unknown) were recorded in the EBCR. Their median age was 72 years (Q1: 57 years, Q3: 80 years, Additional file 1: Table S2). From these patients, a total of 343,284 blood cultures were assessed. In 84.0% (n_BC_ = 288,367) of the blood cultures there was no growth of a pathogen. Overall, 2.8% (n_BC_ = 9571) of blood cultures were classified as contaminated, in 13.2% (n_BC_ = 45,346) of blood cultures at least one relevant pathogen was found (Table 1). Overall, 23,085 BSI events with 28,366 episodes in which a pathogen was detected were recorded (Table 2). *E. coli* was the most commonly detected pathogen (25.4%, n_episode_ = 7207) followed by *Staphylococcus aureus* (15.1%, n_episode_ = 4297). In third, respectively fourth place of relevant pathogens there was S*. epidermidis* (8.1%, n_episode_ = 2309) and *Klebsiella pneumoniae* (4.6%, n_episode_ = 1302). This order did not change during the surveillance period (Table 2). Together, the top ten pathogens accounted for 71.7% of all pathogenic microorganisms in the EBCR during the surveillance period.

**Table 1:** Number of blood cultures, patients and patient demographics per year. BCs: blood cultures

Year	Blood cultures				Patients		
	n_BC_	Negative	Positive	Contaminated	n	Age	Sex
						Mean/Median (Q1; Q3) [years]	(male, %)**
		BCs n_BC_ (%)	BCs n_BC_ (%)	BCs n_BC_ (%)			
2015	50,452	41,852 (83.0)	7296 (14.4)	1304 (2.6)	12,644	63.8 70 (55;79)	57.2
2016	60,483	50,067 (82.8)	8226 (13.6)	2190 (3.6)	18,170	64.7 72 (57;80)	63.0
2017	63,219	51,800 (81.9)	9309 (14.7)	2110 (3.4)	18,742	65.5 73 (58;81)	61.2
2018	83,790	70,938 (84.7)	10,755 (12.8)	2097 (2.5)	21,820	66.3 72 (59;81)	57.2
2019	85,340	73,710 (86.4)	9760 (11.4)	1870 (2.2)	20,291	66.9 72 (59;81)	57.9
total	343,284	288,367 (84.0)	45,346 (13.2)	9571 (2.8)	82,527*	65.2 72 (57;80)	58.7

^*^For total number of patients each patient was only counted once during the 5-year period, **for patients for whom the sex was known

**Table 2:** Distribution of the most commonly pathogens of all detected relevant pathogens in 23,085 events of BSI (n_episode_: number of episodes. % of all episodes in each column); for a definition of “event” and “episode” see main text

Pathogen	overall		2015		2016		2017		2018		2019	
	n_episode_ = 28,366	%	n_episode_ = 4845	%	n_episode_ = 5242	%	n_episode_ = 5722	%	n_episode_ = 6573	%	n_episode_ = 5,985	%
*E. coli*	7207	25.4	1133	23.4	1343	25.6	1490	26.0	1678	25.5	1563	26.1
*S. aureus*	4297	15.2	759	15.7	832	15.9	859	15.0	1005	15.3	842	14.1
*S. epidermidis*	2309	8.1	491	10.1	411	7.8	458	8.0	494	7.5	455	7.6
*K. pneumonia*	1302	4.6	235	4.9	255	4.9	253	4.4	275	4.2	284	4.7
*E. faecalis*	1250	4.4	222	4.6	231	4.4	263	4.6	268	4.1	266	4.4
*E. faecium*	1185	4.2	259	5.3	239	4.6	234	4.1	251	3.8	202	3.4
*Candida spp.*	760	2.7	137	2.8	181	3.5	176	3.1	158	2.4	109	1.8
*P. mirabilis*	636	2.2	89	1.8	105	2.0	120	2.1	169	2.6	153	2.6
*P. aeruginosa*	489	1.7	63	1.3	78	1.5	93	1.6	136	2.1	119	2.0
*S. pneumoniae*	461	1.6	66	1.4	77	1.5	91	1.6	137	2.1	90	1.5
*S. hominis*	447	1.6	123	2.5	60	1.1	80	1.4	93	1.4	91	1.5
other	8023	28.3	1268	26.2	1430	27.3	1605	28.0	1909	29.0	1811	30.2

### Gram-positive pathogens

**Fig. 1:**
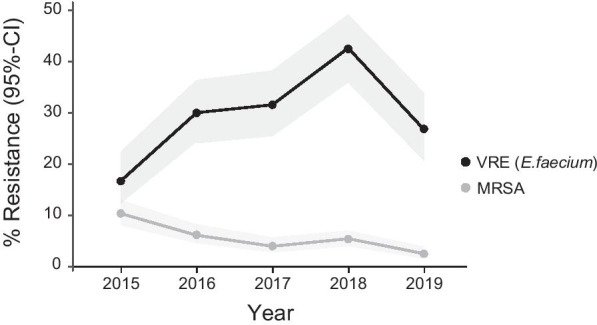
The estimated rate of methicillin resistance in *S. aureus* (●) and vancomycin resistance in *E. faecium* (●) during the surveillance period (cross-sectionally) with 95% confidence interval (95% CI)

During the entire surveillance period 4297 episodes with *S. aureus* and 1185 episodes with *E. faecium* BSI were reported. We observed a decline of oxacillin/methicillin resistance in *S. aureus* (MRSA) from 10.4% in 2015 to 2.5% in 2019 (OR [per year]: 0.74 (95% confidence interval (CI) 0.66–0.83; *p* < 0.001) (Fig. 1, Table 3 and Additional file 1: Table S3). The estimate was robust after adjusting for hospital size (OR_adj_ [per year]: 0.76 (95% confidence interval (CI) 0.68–0.85; *p* < 0.001) (Additional file 1: Table S4). In addition, we also observed evidence for a decline in fluoroquinolones resistance (2015: 31.1%; 2019: 20.1%, *p* < 0.001, (OR [per year]: 0.81 95% CI 0.76–0.87, Table 3, Additional file 1: Table S4) and a stable and low resistance rate for rifampicin (2015: 0.4%; 2019: 0.8%, OR [per year]: 1.24, 95% CI 0.74–2.06, *p* = 0.41) (Table 3) in *S. aureus*.

**Table 3:** *S. aureus* and *E. faecium* susceptibility patterns for selected antibiotics per year

	2015	2016	2017	2018	2019	OR (95% CI) *p* value
*S. aureus*						
Oxacillin/Methicillin	608/63 (10.4)	772/48 (6.2)	818/33 (4.0)	933/50 (5.4)	824/21 (2.5)	0.74 (0.66; 0.83) < 0.001
Rifampicin	550/2 (0.4)	598/4 (0.7)	510/10 (1.9)	735/2 (0.3)	597/5 (0.8)	1.05 (0.81; 1.37) 0.71
Fluoroquinolones	608/190 (31.3)	767/186 (24.3)	813/167 (20.5)	800/144 (18.0)	627/126 (20.1)	0.81 (0.76; 0.87) < 0.001
*E. faecium*						
Vancomycin	227/38 (16.7)	227/68 (30.0)	215/68 (31.6)	228/97 (42.5)	186/50 (26.9)	1.19 (1.08; 1.31) < 0.001
Teicoplanin	186/32 (17.2)	184/49 (26.6)	150/42 (28.0)	196/56 (28.6)	156/22 (14.1)	0.99 (0.88; 1.12) 0.92
Linezolid	220/3 (1.4)	219/2 (0.9)	205/4 (2.0)	222/20 (9.0)	185/5 (2.7)	1.50 (1.19; 1.90) < 0.001
Daptomycin	7/1 (14.3)	16/2 (12.5)	24/2 (8.3)	32/3 (9.4)	48/8 (16.7)	1.08 (0.74; 1.57) 0.71
Tigecycline	149/1 (0.7)	159/1 (0.6)	190/2 (1.1)	213/1 (0.5)	161/6 (3.7)	1.79 (0.89; 3.62) 0.10

Number of tested isolates/number of resistant isolates (% resistant). The right column displays the results from the logistic regression with GEE as odds ratio (OR) for resistance for the unadjusted linear predictor year (i.e. OR per year) with 95% confidence interval (95% CI) and corresponding two-sided *p* value

In contrast, vancomycin resistance rate in *E. faecium* (VRE) increased from 16.7% in 2015 to 26.9% in 2019 (OR 1.19, 95% CI 1.08–1.31, *p* < 0.001), with a distinctive peak in 2018 with a resistance rate of 42.5% (Fig. 1, Table 3, Additional file 1: Table S5 and S6). Since only few isolates have been tested for daptomycin resistance, especially at the beginning of the surveillance, it is difficult to draw conclusions for a change over time. However, resistance rate in 2019 was 16.7% in 48 isolates (Table 2). For tigecycline, resistance rates were low (2015: 0.7%; 2019: 3.7%). Interestingly, 67.9% (218/321) of all VRE episodes recorded during the surveillance period were reported by hospitals with more than 800 beds.

### Gram-negative pathogens

**Fig. 2:**
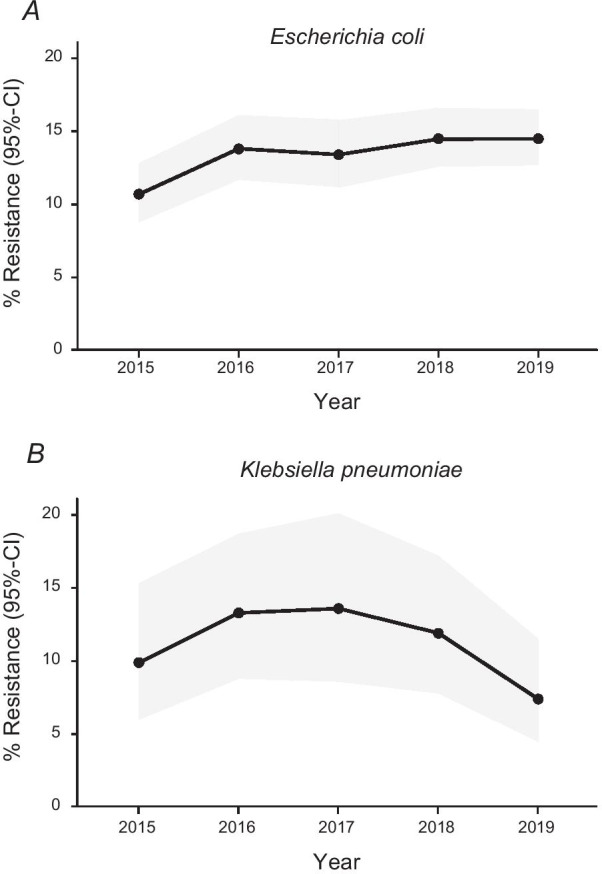
The estimated rate of third-generation cephalosporin resistance (3GC) in *E. coli* (**A**) and *K. pneumoniae* (**B**) during the surveillance period (cross-sectionally) with 95% confidence interval (95% CI)

During the entire surveillance period 7,207 episodes with *E. coli*, 1302 episodes with *K. pneumoniae*, 489 episodes with *Pseudomonas aeruginosa* and 187 episodes with *Acinetobacter spp*. were reported (Table 2). Detailed resistance rates for selected antibiotics per year are displayed in Table 4. Most notably, Cefotaxime resistance in *E. coli* have increased from 10.7% in 2015 to 14.5% in 2019 (OR 1.08, 95% CI 1.02–1.14, *p* = 0.007, Fig. 2A, Table 4 and Additional file 1: Table S7). In contrast, cefotaxime resistance in *K. pneumoniae* was stable (2015: 9.9%; 2019: 7.4%, OR 0.93, 95% CI 0.81–1.08, *p* = 0.35, Fig. 2B, Table 4 and Additional file 1: Table S8). Carbapenem resistance was below 1% in *E. coli* and *K. pneumoniae* and low in *Acinetobacter* spp.*,* with a maximum of 8.6% in 2017. The temporal patterns of antibiotic resistance rates of *E. coli*, *K. pneumonia and Pseudomonas aeruginosa* were robust with regard to adjustment for hospital size (Additional file 1: Tables S7–S9).

**Table 4:** Susceptibility patterns for selected antibiotic substances and pathogens per year

	2015	2016	2017	2018	2019	OR (95% CI) *p* value
*Escherichia coli*						
Aminopenicillin	941/486 (51.6)	1,282/711 (55.5)	1,426/785 (55.0)	1,595/920 (57.7)	1,536/940 (61.2)	1.06 (1.02; 1.10) 0.006
Fluoroquinolones	941/221 (23.5)	1,286/315 (24.5)	1,441/367 (25.5)	1,600/438 (27.4)	1,567/384 (24.5)	1.01 (0.97; 1.05) 0.72
Cefotaxime	938/100 (10.7)	973/134 (13.8)	912/122 (13.4)	1,228/178 (14.5)	1,355/197 (14.5)	1.08 (1.02; 1.14). 0.007
Carbapenems	941/1 (0.1)	1,286/3 (0.2)	1,441/3 (0.2)	1,600/0 (0.0)	1,538/1 (0.1)	–^6^
Aminoglycosides	941/57 (6.1)	1,286/122 (9.5)	1,473/143 (9.7)	1,594/151 (9.5)	1,535/280 (18.2)^2^	1.30 (1.21; 1.40) < 0.001
Fosfomycin	339/1 (0.4)	562/6 (1.1)	630/6 (1.0)	637/9 (1.4)	518/7 (1.4)	–^6^
Trimethoprim-sulfamethoxazole	941/293 (31.1)	1281/408 (31.9)	1435/439 (30.6)	1593/444 (27.9)	1533 /403 (26.3)	0.93 (0.89; 0.97) < 0.001
*Klebsiella pneumoniae*						
Fluoroquinolones	181/25 (13.8)	232/38 (16.4)	227/40 (17.6)	254/49 (19.3)	270/36 (13.3)	1.02 (0.91; 1.15) 0.72
Cefotaxime	181/18 (9.9)	197/26 (13.3)	154/21 (13.6)	202/24 (11.9)	242/18 (7.4)	0.93 (0.81; 1.08) 0.35
Carbapenems	181/2 (1.1)	232/0 (0.0)	227/0 (0.0)	254/2 (0.8)	270/2 (0.7)	–^6^
Aminoglycosides	181/7 (3.9)	232/12 (5.2)	227/14 (6.2)	253/18 (7.1)	270/33 (12.2)^2^	1.47 (1.19; 1.82) < 0.001
Fosfomycin	54/6 (11.1)	117/19 (16.2)	113/15 (13.3)	115/16 (13.9)	92/12 (13.0)	1.01 (0.82; 1.24) 0.95
Trimethoprim-sulfamethoxazole	181/22 (12.2)	231/34 (14.7)	227/33 (14.5)	253/33 (13.0)	270/39 (14.4)	1.06 (0.88; 1.26) 0.56
*Pseudomonas aeruginosa*						
Fluoroquinolones	48/10 (16.3)	72/15 (18.1)	87/23 (16.5)	120/19 (13.5)	–^5^	1.21 (0.97; 1.51) 0.09
Ceftazidime	47/3 (4.8)	72/3 (2.8)	85/7 (7.2)	127/6 (4.0)	–^5^	2.14 (1.29; 3.54) 0.003
Piperacillin/Tazobactam	46/7 (12.2)	71/4 (5.6)	85/9 (9.5)	131/19 (14.1)	–^5^	1.30 (0.97; 1.74) 0.07
Carbapenems	46/4 (9.8)	72/10 (13.9)	88/17 (17.4)	131/17 (11.7)	–^5^	1.17 (0.92; 1.49) 0.20
Aminoglycosides	47/6 (11.9)	72/4 (2.8)	88/8 (7.0)	129/3 (1.6)	–^5^	1.03 (0.72; 1.47) 0.88
Combined resistance^3^	46/5 (10.9)	71/3 (4.2)	87/8 (9.2)	127/7 (5.5)	–^5^	–^6^
*Acinetobacter spp.*					–^5^	
Fluoroquinolones	27/5 (18.5)	33/6 (18.2)	35/11 (31.4)	32/8 (25.0)	–^5^	–^6^
Carbapenems	27/0 (0.0)	33/1 (3.0)	35/3 (8.6)	33/1 (3.0)	–^5^	–^6^
Aminoglycosides	27/1 (3.7)	33/0 (0.0)	33/3 (9.1)	32/1 (3.1)	–^5^	–^6^
Combined resistance^4^	27/0 (0.0)	33/0 (0.0)	33/2 (6.1)	32/1 (3.1)	–^5^	–^6^

Number of tested isolates/number of resistant^1^ isolates (% resistant). The right column displays the results from the logistic regression with GEE as odds ratio (OR) for resistance for the unadjusted linear predictor year (i.e. OR per year) with 95% confidence interval (95% CI) and corresponding two-sided p-value

^1^Resistant and intermediate isolates; number of tested pathogens per year and antimicrobial substance can vary as not always antimicrobial testing for each substance was performed in each isolate, ^2^increase of resistance most probably due to new EUCAST MIC breakpoints, ^3^Combined resistance of at least 3 out of 5 antibiotics under surveillance, ^4^Resistent to all tested antibiotics under surveillance, ^5^not reported due to change in EUCAST methodology, ^6^not evaluated due to the small numbers of event

### Candida

Overall, 760 blood culture episodes with *Candida* spp. were reported during the 5-year surveillance period. *C. albicans* (49.3%, n_episode_ = 375) was most often isolated, followed by *C. glabrata* (33.3%, n_episode_ = 253) and *C. parapsilosis* (6.3%, n_episode_ = 48) (Additional file 1: Table S10).

## Discussion

To the best of our knowledge, this is the largest study on the epidemiology of bloodstream infections in Germany for the last two decades. During the surveillance period a total of 343,284 blood cultures in 82,527 patients were recorded. In 13.2% of blood cultures there was growth of a relevant pathogen, 2.8% of all blood cultures were classified as contaminated. This is in line with the recommendation that blood culture contamination rates should not exceed 3% of blood cultures performed which is considered the standard benchmark [9]. The last study on the epidemiology of BSIs in Germany was published in 2002 [10]. Rosenthal reported 10,052 pathogens from 9555 patients collected in 20 German and two Austrian microbiology labs between September 2000 and August 2001. Compared to our study, the distribution of the five most frequently detected pathogens were similar. *E. coli* was also the most common pathogen at that time (22.6%), followed by *S. aureus* (21.6%), coagulase negative staphylococci (9.2%), *Klebsiella spp*. (6.2%) and Enterococci (8.1%). In addition to the distribution of pathogens in blood cultures, we were also able to describe the course of antimicrobial resistance over a 5-year period.

### MRSA

As recognized previously in many other European countries, we observed a decline in the proportion of methicillin resistance among *S. aureus* isolates [11, 12]. The rate of 5.4% in 2018 is lower than the rate observed in the German Antimicrobial Resistance Surveillance (ARS) of the Robert Koch-Institut (8.0%) [13] and distinctively lower than the European population-weighted mean (16.4%) reported by the European Antimicrobial Resistance Network (EARS-Net) [14]. For both networks, data for 2019 were not available yet.

### VRE

In contrast, Vancomycin resistance in *E. faecium* (VRE) showed an increase with nearly tripling the rate between 2015 and 2018 from 16.7 to 42.5%. A lower rate was observed for 2019, however. The increase was more pronounced than the increase observed in the German ARS network (2015: 12%; 2018: 24%) [13]. This difference might be due to differences in regional occurrence of VRE as suggested by Markwart et al. [15]. The authors analysed routine vancomycin susceptibility testing of 35,906 clinical *E. faecium* isolates from 148 hospitals from 2012 to 2017 using data from the German ARS network. From 2014 onwards the proportions of clinical *E. faecium* isolates exhibiting resistance to vancomycin increased from 11.2 to 26.1% in 2017. The rise of VRE was primarily observed in southern regions of Germany, whereas northern regions did not show a major increase. In the Southwest and Southeast regions—which include Thuringia—VRE proportions increased from 10.8% and 3.8% in 2014 to 36.7% and 36.8% in 2017, respectively. However, it remains unclear, why a large variation in the proportion of VRE exists between German federal states. The temporarily sharp increase of VRE in 2018 observed in our study was accompanied by a likewise sharp increase of Linezolid resistance in *E. faecium* from 2.0% in 2017 to 9.0% in 2018 and back to 2.7% in 2019. In contrast, data from the German ARS network show that the rates for *E. faecium* resistant to Linezolid are small and were constant between 2012 and 2017 (2012: 0.6%; 2017: 0.4%) [16]. This temporary increase in Linezolid resistant VREs observed in our study might reflect a (unnoticed) regional or local outbreak. Indeed, numbers of VRE BSIs showed a disproportionately increase of 28% between 2017 and 2018 in the largest participating hospital, followed by a decline of numbers in 2019. Furthermore, the study shows that about two third of all VRE episodes were reported by hospitals with more than 800 beds. This might reflect the fact that these hospitals offer a specific therapeutic spectrum, including solid organ and/or hematopoietic stemcell transplantation, both known risk factors for VRE [17, 18].

### Gram-negative pathogens

In terms of resistance in gram-negative bacteria, our results are in line with the results of the German ARS network showing that carbapenem resistance in *E. coli* and *K. pneumoniae* is below 1%. Resistance to third-generation cephalosporins (3GC) in *E. coli* increased during the surveillance period (2015: 10.7%; 2018: 14.5%) and was stable in *K. pneumoniae* (2015: 9.9%; 2018: 7.4%). Compared to nationwide observations in the ARS network, the resistance in 3GC in *E. coli* (ARS 2015: 11.5%; 2018: 12.5%) was higher, but lower in *K. pneumoniae* (ARS 2015: 13.0%; 2018: 12.7%). In *P. aeruginosa* resistance rate for carbapenems was between 9.8% in 2015 and 11.7% in 2018 and higher than that reported in ARS (2015: 8.2%; 2018: 5.1%). In contrast, Ceftazidime resistance was lower compared to ARS (2018: 4.0% vs. 9.7%) but similar for Ciprofloxacin (ARS 2018: 12.2% vs. 11.7%).

### Limitations

Our study has several limitations. First of all, it was not possible to establish AlertsNet as a population-based, representative surveillance study including all hospitals in the defined region. Thus, it is not possible to make a powerful statement about the incidence of individual pathogens, e.g. whether there was a change in incidence over time as reported previously for *E. coli* [19]. This is even more the case as surveillance period of each hospital differed and the number of hospitals covered changed from year to year (i.e.; data were not continuously available for each hospital in order to capture a similar collective of patients). As a consequence, the distribution of pathogens and resistance patterns might have been influenced depending on the contributing data providors. Second, antimicrobial susceptibility tests were performed in different clinical microbiology laboratories according to local antibiotic susceptibility testing methods which might have influenced the results as well. Third, blood culture contamination rates might be incorrect, as no clinical chart review was performed to confirm contamination.

## Conclusion

Regarding the distribution of the pathogens, it can be summarized that their ranking has been constant during the last two decades in our geographic region. Concerning antimicrobial resistance, we observed a decline in MRSA rates which might reflect the success of the mandatory infection prevention and control measures for MRSA. In sharp contrast, we could demonstrate a steady increase of VRE rates, mainly driven by large hospitals offering maximum care. Infection prevention and control measures must be taken to prevent a further increase of VRE. In gram-negative bacteria, 3GC resistance in *E. coli* showed an increase over time, carbapenem resistance is currently not a problem in Thuringia.

## Supplementary Information

**Additional file 1.** Supplementary material.

## Data Availability

The datasets used and/or analysed during the current study are available from the corresponding author on reasonable request.

## Abbreviations

ARS: German antimicrobial resistance surveillance
BC: Blood culture
BSI: Bloodstream infection
CI: Confidence intervals
CoNs: Coagulase-negative staphylococci
EARS-Net: European antimicrobial resistance network
EBCR: Electronic blood culture registry
EUCAST: European committee on antimicrobial susceptibility testing
EU/EEA: European union/European economic area
GEE: Generalized estimating equations
IBSC: International bacteremia surveillance collaborative
KISS: German national nosocomial infection surveillance system
LOINC: Logical observation identifiers names and codes
LPSN: List of prokaryotic names with standing in nomenclature
MRSA: Methicillin resistance in *S. aureus*
OR: Odds ratio
PCV: Pneumococcal conjugate vaccine
PPV23: 23-Valent pneumococcal polysaccharide vaccine
VRE: Vancomycin resistant *E. faecium*
