# Supplementary material for: The epidemiology of bloodstream infections and antimicrobial susceptibility patterns in Thuringia, Germany: a five-year prospective, state-wide surveillance study (AlertsNet)
Source: Antimicrob Resist Infect Control. 2021 Sep 8;10:132. doi: 10.1186/s13756-021-00997-6 (PMC8424790; doi:10.1186/s13756-021-00997-6)
Supplement: Supplementary file 1 — Additional file 1. Supplementary material. [file 13756_2021_997_MOESM1_ESM.docx]

**Table S1:** Participating hospitals with corresponding reporting period and hospital size in number of beds. Note that number of beds per hospital may have changed during the whole study period.

| **Hospital** | **Surveillance period** | **Number of hospital beds** |
| --- | --- | --- |
| 1 | 01/15-12/19 | >800 |
| 2 | 02/16-12/19 | <200 |
| 3 | 05/16-12/19 | 401-600 |
| 4 | 07/16-12/19 | 201-400 |
| 5 | 01/15-12/19 | <200 |
| 6 | 02/15-08/16 | 401-600 |
| 7 | 02/15-08/16 | <200 |
| 8 | 02/15-08/16 | 201-400 |
| 9 | 02/15-08/16 | >800 |
| 10 | 03/16-12/19 | 401-600 |
| 11 | 05/16-08/18 | 201-400 |
| 12 | 06/16-08/18 | <200 |
| 13 | 05/16-08/18 | <200 |
| 14 | 05/16-08/18 | 401-600 |
| 15 | 05/16-08/18 | <200 |
| 16 | 08/16-12/19 | <200 |
| 17 | 07/16-12/19 | 201-400 |
| 18 | 01/16-12/19 | <200 |
| 19 | 11/16-02/19 | 601-800 |
| 20 | 06/18-12/19 | >800 |
| 21 | 01/18 | 201-400 |
| 22 | 02/15-12/19 | 401-600 |
| 23 | 01/18 | 401-600 |
| 24 | 11/17-12/19 | 601-800 |
| 25 | 05/19-12/19 | 401-600 |
| 26 | 05/19 | <200 |

**Table S2.** Age distribution of all patients reported to the registry.

| Age (years) | Patients (n) |
| --- | --- |
| 0-9 | 4.166 |
| 10-17 | 823 |
| 18 - < 30 | 2.572 |
| 30 - < 40 | 2.944 |
| 40 - < 49 | 3.687 |
| 50 - < 59 | 8.868 |
| 60 - < 69 | 15.131 |
| 70 - < 79 | 21.126 |
| 80 - < 89 | 19.557 |
| ≥90 | 3.653 |
| total | 82.527 |

**Table S3:** Oxacillin/Methicillin resistance in *S. aureus* isolates during surveillance period and hospital size (r=resistant. s=susceptible), number of isolates (%).

| Hospital size (number of beds) | | 2015 | | | | | | 2016 | | | | | 2017 | | | | | 2018 | | | 2019 | |
| --- | --- | --- | --- | --- | --- | --- | --- | --- | --- | --- | --- | --- | --- | --- | --- | --- | --- | --- | --- | --- | --- | --- |
|  |  | | **s** | | **r** | | **s** | | | **r** | | **s** | | | **r** | | **s** | | | **r** | **s** | **r** |
| ≤ 200 | | 23  (92.0) | | 2  (8.0) | | 98  (97.0) | | | 3  (3.0) | | 196  (99.0) | | | 2  (1.0) | | 159  (98.8) | | | 5  (1.2) | | 107  (97.3) | 3  (2.7) |
| 201-400 | | 71  (89.9) | | 8  (10.1) | | 99  (90.0) | | | 11  (10.0) | | 93  (93.0) | | | 7  (7.0) | | 88  (93.6) | | | 6  (6.4) | | 63  (98.4) | 1  (1.6) |
| 401-600 | | 96  (93.2) | | 7  (6.8) | | 251  (96.2) | | | 10  (3.8) | | 279  (96.9) | | | 9  (3.1) | | 283  (95.0) | | | 15  (5.0) | | 248  (98.4) | 4  (1.6) |
| 601-800 | | - | | - | | 7  (77.8) | | | 2  (22.2) | | 50  (96.2) | | | 2  (3.8) | | 169  (94.4) | | | 10  (5.6) | | 138  (94.5) | 8  (5.5) |
| > 800 | | 355  (88.5) | | 46  (11.5) | | 269  (92.4) | | | 22  (7.6) | | 167  (92.8) | | | 13  (7.2) | | 184  (92.9) | | | 14  (7.1) | | 247  (98.0) | 5  (2.0) |

**Table S4:** Oxacillin/methicillin and fluoroquinolones resistance in *S. aureus* isolates. Results from the logistic regression with GEE as odds ratio (OR) for resistance for an unadjusted or adjusted model for linear predictor year (i.e. OR per year) with 95% confidence interval (95% CI) and corresponding two-sided p-value. The adjusted model also included hospital size (number of beds in categories).

| **models** predictors | **Methicillin/Oxacillin**  OR (95% CI)  p-value | **Fluoroquinolones**  OR (95% CI)  p-value |
| --- | --- | --- |
| unadjusted |  |  |
| time [per year] | 0.74 (0.66; 0.83)  < 0.001 | 0.81 (0.76; 0.87)  < 0.001 |
| adjusted |  |  |
| time [per year] | 0.76 (0.68; 0.85)  < 0.001 | 0.85 (0.79; 0.91)  < 0.001 |
| hospital size [Reference ≤ 200 beds] |  |  |
| 201-400 beds | 2.67 (1.34; 5.34)  0.005 | 1.20 (0.84; 1.74)  0.32 |
| 401-600 beds | 1.45 (0.74; 2.84)  0.28 | 0.93 (0.71; 1.22)  0.60 |
| 601-800 beds | 3.00 (1.42; 6.34)  0.004 | 1.04 (0.73; 1.49)  0.81 |
| > 800 beds | 2.57 (1.38; 4.76)  0.003 | 1.30 (0.99; 1.71)  0.06 |

**Table S5:** Vancomycin resistance in *E. faecium* isolates during surveillance period and hospital size (r=resistant. s=susceptible), number of isolates (%).

| Hospital size (number of beds) | | 2015 | | | | | | 2016 | | | | | 2017 | | | | | 2018 | | | 2019 | |
| --- | --- | --- | --- | --- | --- | --- | --- | --- | --- | --- | --- | --- | --- | --- | --- | --- | --- | --- | --- | --- | --- | --- |
|  |  | | **s** | | **r** | | **s** | | | **r** | | **s** | | | **r** | | **s** | | | **r** | **s** | **r** |
| ≤ 200 | | 11  (100) | | 0  (0.0) | | 12  (66.7) | | | 6  (33.3) | | 16  (88.9) | | | 2  (11.1) | | 13  (86.7) | | | 2  (13.3) | | 7  (77.8) | 2  (22.2) |
| 201-400 | | 11  (84.6) | | 2  (15.4) | | 10  (66.7) | | | 5  (33.3) | | 9  (39.1) | | | 14  (60.9) | | 5  (83.3) | | | 1  (16.7) | | 3  (100) | 0  (0) |
| 401-600 | | 21  (84.0) | | 4  (16.0) | | 31  (88.6) | | | 4  (11.4) | | 44  (86.3) | | | 7  (13.7) | | 18  (52.9) | | | 16  (47.1) | | 23  (62.2) | 14  (37.8) |
| 601-800 | | - | | - | | 3  (42.9) | | | 4  (57.1) | | 11  (55.0) | | | 9  (45.0) | | 32  (82.2) | | | 6  (17.8) | | 18  (78.3) | 5  (21.7) |
| > 800 | | 146  (82.0) | | 32  (18.0) | | 103  (67.8) | | | 49  (32.2) | | 67  (65.0) | | | 36  (35.0) | | 63  (43.4) | | | 72  (56.6) | | 85  (74.6) | 29  (25.4) |

**Table S6:** Vancomycin and teicoplanin resistance in *E. faecium* isolates. Results from the logistic regression with GEE as odds ratio (OR) for resistance for an unadjusted or adjusted model for linear predictor year (i.e. OR per year) with 95% confidence interval (95% CI) and corresponding two-sided p-value. The adjusted model also included hospital size (number of beds in categories).

| **models** predictors | **Vancomycin**  OR (95% CI)  p-value | **Teicoplanin**  OR (95% CI)  p-value |
| --- | --- | --- |
| unadjusted |  |  |
| time [per year] | 1.19 (1.08; 1.31)  < 0.001 | 0.99 (0.88; 1.12)  0.92 |
| adjusted |  |  |
| time [per year] | 1.23 (1.11; 1.36)  < 0.001 | 1.03 (0.91; 1.16)  0.67 |
| hospital size [Reference ≤ 200 beds] |  |  |
| 201-400 beds | 3.13 (1.22; 8.05)  0.02 | 3.31 (0.57; 19.19)  0.18 |
| 401-600 beds | 1.55 (0.75; 3.19)  0.24 | 1.04 (0.41; 2.61)  0.93 |
| 601-800 beds | 1.53 (0.64; 4.68)  0.34 | 0.68 (0.26; 1.78)  0.44 |
| > 800 beds | 2.38 (1.26; 4.41)  0.008 | 1.29 (0.60; 2.77)  0.51 |

**Table S7*:*** *E. coli* resistance rates of selected antibiotics. Results from the logistic regression with GEE as odds ratio (OR) for resistance for an unadjusted or adjusted model for linear predictor year (i.e. OR per year) with 95% confidence interval (95% CI) and corresponding two-sided p-value. The adjusted model also included hospital size (number of beds in categories).

| models  predictors | Aminopenicilline OR (95% CI) p-value | Fluoroquinolones OR (95% CI) p-value | Aminoglycosides OR (95% CI) p-value | Cefotaxime OR (95% CI) p-value | Trimethoprim/ Sulfamethoxazole OR (95% CI) p-value |
| --- | --- | --- | --- | --- | --- |
| unadjusted  time [per year] | 1.06 (1.02; 1.10)  0.006 | 1.01 (0.97; 1.05)  0.72 | 1.30 (1.21; 1.40)  < 0.001 | 1.08 (1.02; 1.14)  0.007 | 0.93 (0.89; 0.97)  < 0.001 |
| adjusted  time [per year] | 1.07 (1.03; 1.12)  < 0.001 | 1.02 (0.98; 1.06)  0.39 | 1.33 (1.23; 1.44)  < 0.001 | 1.09 (1.03; 1.15)  0.004 | 0.94 (0.90; 0.98)  0.003 |
| hospital size  [Reference ≤ 200 beds] |  |  |  |  |  |
| 201-400 beds | 0.81 (0.66; 0.99)  0.04 | 1.15 (0.90; 1.47)  0.25 | 0.82 (0.59; 1.12)  0.21 | 1.01 (0.67; 1.52)  0.96 | 0.91 (0.73; 1.14)  0.42 |
| 401-600 beds | 1.07 (0.91; 1.26)  0.39 | 1.49 (1.23; 1.81)  < 0.001 | 0.77 (0.60; 0.98)  0.04 | 0.92 (0.67; 1.27)  0.62 | 1.07 (0.90; 1.27)  0.45 |
| 601-800 beds | 0.60 (0.49; 0.75)  < 0.001 | 1.03 (0.78; 1.35)  0.86 | 0.38 (0.26; 0.57)  < 0.001 | 0.96 (0.64; 1.45)  0.85 | 1.01 (0.79; 1.29)  0.93 |
| > 800 beds | 1.09 (0.93; 1.28)  0.28 | 1.58 (1.31; 1.90)  < 0.001 | 0.58 (0.45; 0.75)  < 0.001 | 1.10 (0.81; 1.49)  0.55 | 1.39 (1.18; 1.65)  < 0.001 |

**Table S8*:*** *K. pneumoniae* resistance rates of selected antibiotics. Results from the logistic regression with GEE as odds ratio (OR) for resistance for an unadjusted or adjusted model for linear predictor year (i.e. OR per year) with 95% confidence interval (95% CI) and corresponding two-sided p-value. The adjusted model also included hospital size (number of beds in categories).

| models  predictors | Aminoglycosides  OR (95% CI) p-value | Cefotaxime  OR (95% CI) p-value | Fluoroquinolones  OR (95% CI) p-value | Fosfomycin  OR (95% CI) p-value | Trimethoprim/ Sulfamethoxazole  OR (95% CI) p-value |
| --- | --- | --- | --- | --- | --- |
| unadjusted  time [per year] | 1.47 (1.19; 1.89)  <0.001 | 0.93 (0.81; 1.08)  0.35 | 1.02 (0.91; 1.15)  0.75 | 1.01 (0.82; 1.24)  0.95 | 1.02 (0.89; 1.17)  0.75 |
| adjusted  time [per year] | 1.48 (1.19; 1.86)  <0.001 | 0.97 (0.84; 1.11)  0.65 | 1.03 (0.91; 1.16)  0.64 | 1.06 (0.82; 1.36)  0.68 | 1.03 (0.89; 1.18)  0.73 |
| hospital size  [Reference ≤ 200 beds] |  |  |  |  |  |
| 201-400 beds | 0.29 (0.06; 1.40)  0.12 | 1.13 (0.42; 3.04)  0.80 | 1.54 (0.64; 3.67)  0.33 | 1.11 (0.31; 3.93)  0.87 | 1.11 (0.46; 2.67)  0.82 |
| 401-600 beds | 0.95 (0.43; 2.12)  0.91 | 0.39 (0.17; 0.89)  0.03 | 1.31 (0.63; 2.72)  0.47 | 1.13 (0.49; 2.59)  0.78 | 0.92 (0.46; 1.84)  0.82 |
| 601-800 beds | 0.45 (0.15; 1.35)  0.15 | 0.51 (0.17; 1.49)  0.22 | 1.43 (0.63; 3.22)  0.39 | 1.14 (0.41; 3.13)  0.80 | 1.34 (0.63; 2.85)  0.44 |
| > 800 beds | 0.55 (0.24; 1.23)  0.14 | 0.66 (0.31; 1.39)  0.28 | 1.57 (0.78; 3.14)  0.21 | 1.69 (0.57; 4.97)  0.34 | 1.40 (0.73; 2.68)  0.31 |

**Table S9*:*** *P. aeruginosa* resistance rates of selected antibiotics. Results from the logistic regression with GEE as odds ratio (OR) for resistance for an unadjusted or adjusted model for linear predictor year (i.e. OR per year) with 95% confidence interval (95% CI) and corresponding two-sided p-value. The adjusted model also included hospital size (number of beds in categories).

| models  predictors | Fluoroquinolones  OR (95% CI) p-value | Piparacillin/Tazobactam  OR (95% CI) p-value |
| --- | --- | --- |
| unadjusted  time [per year] | 1.21 (0.97; 1.51)  0.09 | 1.30 (0.97; 1.74)  0.07 |
| adjusted  time [per year] | 1.21 (0.95; 1.53)  0.12 | 1.31 (0.99; 1.73)  0.06 |
| hospital size   [Reference ≤ 200 beds] |  |  |
| 201-400 beds | 0.20 (0.04; 0.99)  0.05 | 1.23 (0.33; 4.63)  0.76 |
| 401-600 beds | 0.81 (0.33; 1.94)  0.63 | 0.89 (0.29; 2.75)  0.84 |
| 601-800 beds | 1.21 (0.42; 3.46)  0.72 | 1.00 (0.28; 3.66)  0.99 |
| > 800 beds | 0.62 (0.26; 1.44)  0.26 | 1.48 (0.52; 4.19)  0.46 |

**Table S10.** BC episodes with *Candida species* per year under surveillance.

| *Candida sepcies* | 2015 | 2016 | 2017 | 2018 | 2019 |
| --- | --- | --- | --- | --- | --- |
| *Candida albicans* | 60 | 89 | 98 | 79 | 49 |
| *Candida dubliniensis* | 1 | 0 | 0 | 1 | 1 |
| *Candida famata* | 0 | 0 | 1 | 0 | 0 |
| *Candida glabrata* | 48 | 58 | 49 | 56 | 42 |
| *Candida guillermondii* | 4 | 0 | 1 | 0 | 0 |
| *Candida kefyr* | 2 | 6 | 4 | 0 | 0 |
| *Candida krusei* | 8 | 3 | 2 | 0 | 1 |
| *Candida lipolytica* | 2 | 0 | 0 | 0 | 1 |
| *Candida lusitaniae* | 0 | 1 | 5 | 0 | 0 |
| *Candida parapsilosis* | 10 | 14 | 11 | 8 | 5 |
| *Candida rugosa* | 0 | 0 | 0 | 0 | 2 |
| *Candida sphaerica* | 0 | 3 | 0 | 0 | 0 |
| *Candida tropicalis* | 2 | 4 | 4 | 12 | 1 |
| *Candida utilis* | 0 | 0 | 0 | 0 | 1 |
| total | 136 | 178 | 175 | 156 | 103 |
